# Exploring the impact of prior spontaneous miscarriage on stress among pregnant women during the first trimester: an observational study

**DOI:** 10.3399/BJGPO.2022.0100

**Published:** 2023-01-25

**Authors:** Coralie Barbe, Justine Ouy, Marie Boiteux-Chabrier, Leïla Bouazzi, Bach-Nga Pham, Sandra Carrau-Truillet, Aline Hurtaud

**Affiliations:** 1 Comité Universitaire de Ressources pour la Recherche en Santé, Université de Reims Champagne-Ardenne UFR Médecine, Reims, France; 2 Laboratoire C2S (Cognition, Santé, Société), Université de Reims Champagne Ardenne, Reims, France; 3 Département de Médecine Générale, Université de Reims Champagne-Ardenne, UFR Médecine, Reims, France

**Keywords:** pregnancy trimester, first, stress, spontaneous miscarriage, delivery of health care, cross-sectional studies

## Abstract

**Background:**

Spontaneous miscarriage (SM) is the most common complication of pregnancy. Its psychological repercussions are widely documented but few studies have investigated its effect on women’s experience of a subsequent pregnancy.

**Aim:**

To evaluate the impact of prior SM on the level of stress experienced by pregnant women during the first trimester of pregnancy.

**Design and setting:**

Cross-sectional, observational study, which was conducted between June and October 2021 in France.

**Method:**

A self-report questionnaire was distributed to women in the first trimester of pregnancy. Stress was assessed using the Antenatal Perceived Stress Inventory to yield an overall score and a score for three dimensions ('medical and obstetric risks or fetal health'; 'psychosocial changes during pregnancy'; and the ‘prospect of childbirth'). Women with a history of prior SM and those without were compared.

**Results:**

In total, 93 women were included; 63 without and 30 with a history of prior SM. Prior SM was not associated with the overall score. The score for the dimension 'medical and obstetric risks or fetal health' was significantly higher in women with prior SM (3.00±0.86 versus 2.34±0.80; *β* = 0.61 [95% confidence interval {CI} = 0.25 to 0.96]; *P* = 0.001). Prior SM was significantly associated with the items 'the baby’s health' (*P* = 0.048) and 'the echography' (*P* = 0.002).

**Conclusion:**

This study shows a significant impact of prior SM on the level of stress of pregnant women during the first trimester, particularly relating to the medical and obstetric risks or fetal health, underlining the need for appropriate psychological support to be provided to women who experience SM.

## How this fits in

SM, which is the most common complication of early pregnancy, is not a minor event in a woman’s life. Women who have experienced prior SM have levels of stress related to the medical and obstetric risks or fetal health higher than women without prior SM during the first trimester (that is, during the first 12 weeks of amenorrhoea) of a subsequent pregnancy. Appropriate psychological support should be provided to women who experience SM.

## Introduction

SM is defined by the World Health Organization as spontaneous pregnancy loss before the fetus reaches viability, or fixed at 22 weeks of amenorrhoea or a fetal weight of 500 g.^1^ SM is the most common complication of early pregnancy and occurs in 23%–25% of pregnancies, corresponding to around 200 000 cases per year in France and 23 million cases worldwide.^2,3^ SM is often related to anomalies of genetics or embryo development (around 60% of cases), or to maternal factors (for example, uterine anomalies and infectious, immune, or endocrine diseases).^2^ Several risk factors have been shown to play a role in the occurrence of SM, such as maternal age >35 years, or paternal age >40 years, maternal overweight, African-American ethnic group, smoking, consumption of coffee or alcohol, exposure to ionising radiation or pesticides, maternal stress, and a history of prior SM or elective termination of pregnancy.^2,4–6^

Given its frequency, SM may be considered as a minor event by healthcare providers and society in general. Consequently, women tend not to talk about SM, or may even hide its occurrence and any related complications from their healthcare providers, their family, and even their partner.^2^ Yet, numerous studies have reported the psychological impact of SM, and shown it to be a traumatic event that can be experienced as a form of bereavement, with medium- to long-term consequences including sorrow, depression, anxiety, stress, and post-traumatic stress disorder, consequences which may persist up to 2 years after the event.^7,8^ However, few studies have investigated the psychological repercussions of SM on the woman’s life experience, specifically their experience of a subsequent pregnancy. The few available qualitative studies, such as the study by de Montigny *et al*, have reported that the woman’s next pregnancy is likely to be experienced in a climate of apprehension and worry, especially during the first trimester, which is seen as a milestone that they need to pass.^7,9^

Other studies have investigated the effects of maternal stress on the embryo-fetal development and early infancy.^5,10,11^ A higher level of maternal stress was found to be associated with increased fetal heart rate and reduced fetal movements. During the first trimester it was found to be associated with an increased risk of SM. During the first and the second trimester a higher level of maternal stress was found to be associated with premature birth and low birthweight. In infants, maternal stress during the third trimester is associated with more crying, agitation, negative emotional reactions, difficulties with adaptation and attention, and impaired psychomotor development. In the longer term, retarded mental development, psychiatric diseases, and attention deficit hyperactivity disorder may count among the potential consequences of prenatal material stress.

The main aim of this study was to evaluate the impact of a history of SM on the level of stress of women during the first trimester of a subsequent pregnancy. The secondary objective was to describe the level of stress among women during the first trimester of pregnancy.

## Method

### Study design and population

A prospective, multicentre, cross-sectional, observational study comparing women with at least one history of SM versus without history of SM was performed. Participants were recruited from French medical laboratories from June–October 2021. In France, plasma dosage of human chorionic gonadotropin is used in routine care to affirm the beginning of a pregnancy. Women aged ≥18 years, who had a positive plasma dosage of human chorionic gonadotropin, and who were estimated to be in the first trimester according to the date of their last period as reported by the participant (that is, during the first 12 weeks of amenorrhoea) were included. The following women were excluded: those who were already in the second or third trimester as per the estimated delivery date according to the self-reported date of the last period; women being followed for medically assisted fertility; women who had human chorionic gonadotropin testing to document declining human chorionic gonadotropin levels in the context of follow-up after elective pregnancy termination or SM; women who did not intend to pursue the pregnancy to its term; and women who refused consent to participate.

### Data recorded

A self-report questionnaire was provided by email, a maximum of 10 days after the positive plasma dosage of human chorionic gonadotropin test. The questionnaire included 11 questions covering the following: woman’s age, history of prior interrupted pregnancies (SM; voluntary interruption of pregnancy; termination of pregnancy for medical reasons; fetal death in utero; and/or ectopic pregnancy); date of last period; and the intention to pursue the current pregnancy to its term. Seven questions asked about sources of stress during pregnancy, as established in the literature,^12–15^ namely the following: level of education and socioeconomic vulnerability; number of previous pregnancies (gravidity); previous number of deliveries (parity); marital difficulties; social or familial isolation; and whether the current pregnancy was unexpected or unplanned.

Stress was measured using the Antenatal Perceived Stress Inventory (APSI) developed in 2014 and validated in the French language for the evaluation of prenatal stress during the three trimesters of pregnancy.^16^ The APSI is composed of 12 items, all ranked on a five-point Likert scale (not at all, a little, fairly, very, and extremely stressed). The scale yields an overall score and a score for each of the three dimensions, namely the following: 'medical and obstetric risks or fetal health' (including the following items: the baby’s health; screening tests for trisomy; the echography; and obstetric or medical problems I might encounter during pregnancy); 'psychosocial changes during pregnancy' (including the following items: my state of fatigue [current]; my mood swings; my hypersensitivity; and my relationship with my partner); and the ‘prospect of childbirth' (including the following items: the risk of suffering an episiotomy; the prospect or not of having an epidural; having gained weight during pregnancy; not being able to do what I used to do before [such as smoking, drinking, going out, travelling, and sports]; and not knowing the delivery date). The scores are obtained by summing the item scores and then dividing them by the number of items. The higher the score, the higher the stress perceived by the pregnant woman.

### Ethical considerations

All women provided consent to participate. All recorded data were anonymous. Data management was in compliance with current French legislation governing nominative personal data, the General Data Protection Regulation (GDPR) of the European Union, and the The French Data Processing, Data Files and Individual Liberties Act of 6 January 1978 and its modification in 2018.

### Statistical analysis

The sample size was calculated using nQuery software (version 7.0) based on the following assumptions: a mean total APSI score of 2.18±0.56 among women without prior SM, based on the study by Razurel *et al*;^16^ and an expected mean total APSI score of 2.68 among women with a history of at least one prior SM, an alpha risk of 5%, power of 90%, in a bilateral situation. A total of 28 women per group were estimated to be required.

Data are described as mean±standard deviation, median and range, or number and percentage. Factors significantly associated with the stress levels overall and in each dimension of the APSI were studied by univariate analyses using the Student's *t*, Wilcoxon, χ^2^, or Fisher’s exact tests, as appropriate. Variables with a *P*-value <0.10 by univariate analysis were included in multiple linear regression models. Beta coefficients and 95% confidence intervals (CIs) are reported as measures of association. A *P*-value <0.05 was considered statistically significant. Statistical analyses were performed using SAS (version 9.4).

## Results

### Characteristics of the study population

In total, 138 pregnant women received the self-report questionnaire by email. Among them, 93 women responded (67.4%); 63 (67.7%) had never experienced SM, while 30 (32.3%) reported a history of at least one SM. The characteristics of the study population are displayed in Table 1. Average age was 30.0±4.4 years. Most of the women (*n* = 85, 91.4%) were employed, with an educational level of high-school diploma or higher in 92.5% (*n* = 86). The vast majority were married or living with a partner (*n* = 88, 94.6%). Overall, 53.8% (*n* = 50) were nulliparous, 81.7% (*n* = 76) reported that they had adequate support from their family and/or friends, and in 80.6% (*n* = 75) the current pregnancy was expected or planned. There were no significant differences between women with versus without prior SM, except for the number of previous pregnancies (median 1 [range 0–4] for women without versus median 2 [range 1–6] for women with prior SM, *P*<0.001).

**Table 1.** Characteristics of the 93 pregnant women in the first trimester who participated in the study

Variable^a^	Overall(*n* = 93)	Prior SM(*n* = 30)	No prior SM(*n* = 63)	*P* value
Age, years (mean±SD)	30.0±4.4	29.2±4.2	30.3±4.5	0.27
High-school diploma or higher				0.09
Yes	86 (92.5)	30 (100.0)	56 (88.9)	
No	7 (7.5)	0 (0.0)	7 (11.1)	
Professional status				0.92
Employed	85 (91.4)	29 (96.7)	56 (88.9)	
Unemployed	2 (2.2)	0 (0.0)	2 (3.2)	
Stay-home mother	4 (4.3)	1 (3.3)	3 (4.8)	
Student or in training	1 (1.1)	0 (0.0)	1 (1.6)	
Other	1 (1.1)	0 (0.0)	1 (1.6)	
Marital status				1.00
Single	5 (5.4)	1 (3.3)	4 (6.3)	
Married or living maritally	88 (94.6)	29 (96.7)	59 (93.7)	
Number of pregnancies, median (range)	1 (0–6)	2 (1–6)	1 (0–4)	<0.001
Nulliparous				0.20
Yes	50 (53.8)	19 (63.3)	31 (49.2)	
No	43 (46.2)	11 (36.7)	32 (50.8)	
Number of children, median (range)	0 (0–3)	0 (0–3)	1 (0–3)	0.33
Social or familial isolation				0.84
Support available from close family or friends	76 (81.7)	25 (83.3)	51 (81.0)	
Alone and isolated	9 (9.7)	2 (6.7)	7 (11.1)	
Other	8 (8.6)	3 (10.0)	5 (7.9)	
Type of pregnancy				0.40
Expected or planned	75 (80.6)	26 (86.7)	49 (77.8)	
Unexpected or unplanned	18 (19.4)	4 (13.3)	14 (22.2)	
Other history of pregnancy termination^b^				0.96
Yes	22 (23.7)	7 (23.3)	15 (23.8)	
No	71 (76.3)	23 (76.7)	48 (76.2)	

^a^Data are presented as number (%) unless otherwise indicated. ^b^Encompasses voluntary interruption of pregnancy; termination of pregnancy for medical reasons; fetal death in utero; and ectopic pregnancy. SD = standard deviation. SM = spontaneous miscarriage.

### Stress levels and associated factors

The stress levels of the study participants are described in Table 2. The mean overall APSI score was 1.98±0.52, and the scores for the individual dimensions were 2.55±0.87 for medical and obstetric risks or fetal health; 1.68±0.87 for psychosocial changes during pregnancy; and 1.70±0.66 for the prospect of childbirth.

**Table 2.** Description of maternal stress among pregnant women in the first trimester as evaluated by the Antenatal Perceived Stress Inventory (overall score and for each of the three dimensions)

Variables^a^	Overall (*n* = 93)
'Medical and obstetric risks or fetal health' dimension	2.55±0.87
'Psychosocial changes during pregnancy' dimension	1.68±0.87
'Prospect of childbirth' dimension	1.70±0.66
Overall score	1.98±0.52

^a^Mean±standard deviation.

Table 3 presents the univariate analysis of the factors associated with the overall APSI score. The overall score was not significantly associated with a history of prior SM (*P* = 0.34). Nulliparous women had significantly higher overall APSI score (2.12±0.53 versus 1.81±0.46 in women with prior delivery; *P* = 0.004). None of the other variables were associated with overall APSI score and therefore no multivariate analysis was performed.

**Table 3.** Univariate analysis of the factors associated with maternal stress among pregnant women (in the first trimester of pregnancy) as evaluated by the Antenatal Perceived Stress Inventory (overall score and for each of the three dimensions)

Variables^a^	Overall score	*P* value	'Medical and obstetric risks or fetal health' dimension	*P* value	'Psychosocial changes during pregnancy' dimension	*P* value	'Prospect of childbirth' dimension	*P* value
History of prior spontaneous miscarriage		0.34		<0.001		0.05		0.58
Yes (*n* = 30)	2.05±0.46		3.00±0.86		1.48±0.53		1.64±0.64	
No (*n* = 63)	1.94±0.55		2.34±0.80		1.78±0.98		1.72±0.67	
High-school diploma or higher		0.09		0.79		0.01		0.17
Yes (*n* = 86)	1.95±0.51		2.56±0.89		1.62±0.76		1.67±0.63	
No (*n* = 7)	2.30±0.64		2.46±0.53		2.52±1.57		2.03±0.96	
Nulliparous		0.004		0.02		0.65		0.002
Yes (*n* = 50)	2.12±0.53		2.76±0.88		1.65±0.82		1.89±0.71	
No (*n* = 43)	1.81±0.46		2.31±0.80		1.73±0.93		1.47±0.51	
Social or familial isolation^b^		0.27		0.34		0.18		0.37
Support available from close family or friends (*n* = 76)	1.95±0.51		2.53±0.88		1.59±0.71		1.70±0.67	
Alone and isolated (*n* = 9)	2.16±0.69		2.83±1.01		2.37±1.59		1.49±0.62	
Type of pregnancy		0.78		0.62		0.43		0.80
Expected or planned (*n* = 75)	1.97±0.49		2.57±0.86		1.64±0.79		1.69±0.63	
Unexpected or unplanned (*n* = 18)	2.01±0.64		2.46±0.92		1.87±1.15		1.73±0.78	
Other history of pregnancy termination^c^		0.61		0.65		0.41		0.29
Yes (*n* = 22)	1.93±0.47		2.48±0.81		1.82±0.93		1.55±0.55	
No (*n* = 71)	1.99±0.54		2.57±0.89		1.64±0.85		1.74±0.68	

^a^Mean±standard deviation. ^b^For these analyses, ‘Other’ was not included because women who answered ‘Other’ had not yet announced their pregnancy and so could not assess their isolation. ^c^Encompasses voluntary interruption of pregnancy; termination of pregnancy for medical reasons; fetal death in utero; and ectopic pregnancy.

**Figure 1.**
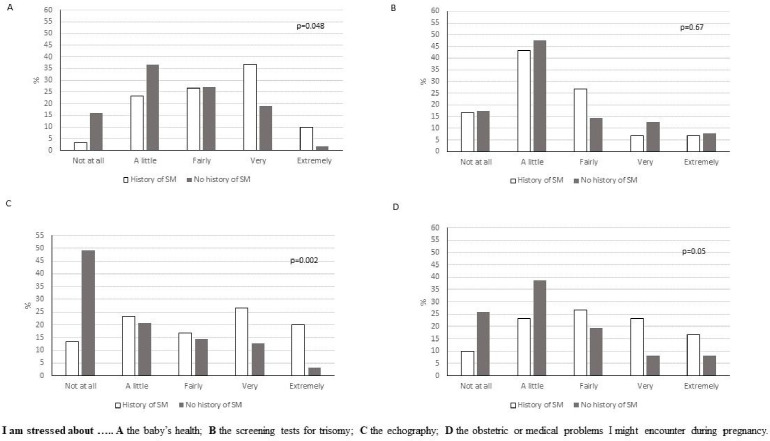
Responses of pregnant women (in the first trimester) on the four items constituting the dimension 'medical and obstetric risks or fetal health' of the Antenatal Perceived Stress Inventory, according to the presence or absence of prior spontaneous miscarriage

Regarding the dimension 'medical and obstetric risks or fetal health', the results of the univariate analysis are presented in Table 3. Higher scores on this dimension were associated with a history of prior SM (*P*<0.001) and nulliparous status (*P* = 0.02). By multivariate analysis, women with a history of SM (3.00±0.86 versus 2.34±0.80 in those without; *β* = 0.61 [95% CI = 0.25 to 0.96]; *P*<0.001) and nulliparous women (2.76±0.88 versus 2.31±0.80 in women with prior delivery; *β* = 0.38 [95% CI = 0.04 to 0.71]; *P* = 0.03) had significantly higher stress scores on this dimension (Table 4). The responses for each item in the 'medical and obstetric risks or fetal health' dimension are detailed in Figure 1 according to the history of prior SM. The responses to the items 'the baby’s health' and 'the echography' differed significantly between women with versus without prior SM (*P* = 0.048 and 0.002, respectively). The item 'the obstetric or medical problems I might encounter during pregnancy' was also borderline significantly different between groups (*P* = 0.05).

**Table 4.** Multivariate analysis of the factors associated with maternal stress among pregnant women (in the first trimester of pregnancy) as evaluated by the Antenatal Perceived Stress Inventory

Dimension	β (95% CI)	*P* value
**'Medical and obstetric risks or fetal health'**		
History of prior spontaneous miscarriage		0.001
Yes	0.61 (0.25 to 0.96)	
No	1	
Nulliparous		0.03
Yes	0.38 (0.04 to 0.71)	
No	1	
**'Psychosocial changes during pregnancy'**		
History of prior spontaneous miscarriage		0.27
Yes	–0.21 (–0.59 to 0.16)	
No	1	
High-school diploma or higher		0.01
Yes	–0.83 (–1.50 to –0.16)	
No	1	

Regarding the dimension 'psychosocial changes during pregnancy' of the APSI scale, the results of the univariate analysis are shown in Table 3. Higher scores on this scale were associated with the level of education (*P* = 0.01) and borderline with the history of SM (*P* = 0.05). By multivariate analysis (Table 4), women with high-school diploma or higher education (1.62±0.76 versus 2.52±1.57; *β* = −0.83 (95% CI = –1.50 to –0.16); *P* = 0.01) were significantly less likely to have higher stress scores on the 'psychosocial changes during pregnancy' dimension, while the history of SM was not significantly associated with stress scores in this subscale (1.48±0.53 versus 1.78±0.98; *β* = −0.21 (95% CI = –0.59 to 0.16); *P* = 0.27).

Regarding the 'prospect of childbirth' dimension, the results of the univariate analysis are presented in Table 3. Higher scores on this dimension were associated with nulliparous status (*P* = 0.002). None of the other variables were associated with this dimension and therefore no multivariate analysis was performed.

## Discussion

### Summary

This study reveals higher levels of stress related to medical and obstetric risks and fetal health in women with a history of prior SM, and in nulliparous women. With regard to the history of SM, the results failed to show a significant association between the overall score on the APSI scale and a history of SM (*P* = 0.34). This could be explained by the fact that the items including the two other dimensions of the APSI (namely, 'psychosocial changes during pregnancy' and 'prospect of childbirth') do not reflect the baby’s health. Conversely, women with a history of SM had significantly higher stress levels on the medical and obstetric risks or fetal health dimension (*P* = <0.001). SM during the first trimester is a common obstetrical complication that involves the death of the fetus.^1^ It is logical that medico-obstetrical risks and the health of the fetus should therefore be a source of stress for a pregnant woman during the first trimester of a subsequent pregnancy after a prior experience of SM.

### Strengths and limitations

To the best of the authors' knowledge, this is the first study to specifically explore the association between maternal stress during the first trimester of pregnancy and a personal history of SM. The multicentre, prospective study used a rigorous methodology, with a calculation *a priori* of the sample size, and established risk factors from the literature were taken into account, notably obstetrical antecedents, such as voluntary or medical termination of pregnancy, enabling the study to highlight the specific effect of prior SM. The number of pregnancies was significantly higher among women with a history of SM (*P*<0.001). SM counts as a pregnancy, even if the pregnancy terminates very prematurely.^1^ Thus, it is congruent that the women with prior SM would have a higher total number of pregnancies than those without a history of SM. The study population included a higher proportion of women with prior SM (32.3%) than rates reported in the general population (25%).^3,4^ This study may have selected women who feel more particularly affected by the subject of SM and concerned by their current pregnancy. However, this higher proportion is not a bias for a case-control study.

### Comparison with existing literature

More specifically, the items associated with higher stress levels were those relating to the baby’s health (*P* = 0.048) and the echography (*P* = 0.002), with a borderline significant difference also observed for the item relating to the potential medical or obstetrical problems that might be encountered during pregnancy (*P* = 0.05). Echography is the gold standard for diagnosing ongoing pregnancy.^6,17^ Performed at the end of the first trimester, it is eagerly awaited by the future mother, who is keen to see the baby move and hear its heartbeat, the markers of a viable pregnancy.^7,9^ However, the ultrasound can also reveal malformations or intrauterine death,^18^ and is thus a source of stress for women who have a history of SM and who dread experiencing another one. In this regard, the items relating to the baby’s health and the potential medical or obstetrical problems that could be encountered during pregnancy, suggestive of a possible problem with the fetus or fetal development that might lead to SM, attest a state of stress among women who have previously experienced SM.^7^

Regarding the level of education, the results show that women who have achieved a high-school diploma or higher had lower levels of stress compared with women with a lower educational level regarding the psychosocial changes during pregnancy (*P* = 0.01). A possible hypothesis to explain this finding is that lower levels of education are often the marker of greater financial insecurity, characterised by lower income, as shown in a survey performed in 2016.^19^ This can be a source of stress for the mother, with the worry that she may not be able to provide for the baby or pay the medical costs that lie ahead.^20^

It was also observed that nulliparous women were significantly more stressed than women who had previously experienced at least one delivery, with regard to the dimensions 'medical and obstetrical risks or fetal health' (*P* = 0.02) and the 'prospect of childbirth' (*P* = 0.002). These findings are concordant with other studies in the literature showing that parity affects the experience of pregnancy.^12,21^ Nulliparous women are more desirous of medical support and depend more on healthcare providers, with a greater need for support. Furthermore, it has been shown that nulliparous women fear delivery, particularly in relation with their perception of the pain of a vaginal birth.^21,22^

### Implications for research and practice

In their classic work published in 1984, Lazarus and Folkman define stress as a *'particular relationship between the person and the environment that is appraised by the person as taxing or exceeding his or her resources and endangering his or her wellbeing'*.^23^ Although clearly not all women who have had SM will require medical management of stress during a subsequent pregnancy, the results of the study underline that SM should not be minimised as it may be traumatic for the woman. The study by Séjourné *et al* has shown the utility of early intervention after SM to provide support and education, and to screen for potential psychological complications that may require appropriate management and follow-up.^24^

It therefore seems important to assess prior experience of SM among pregnant women with a history of prior SM to screen for possible signs of psychological distress, notably stress, in view of the complications that maternal stress may have on the pregnancy and fetus, even into infancy.^10,11^ Indeed, it has been shown that psychological repercussions, such as anxiety or depression, have been observed after SM.^23,24^ Tailored support could be envisaged for women with high levels of stress or anxiety during the first trimester of pregnancy or for those with depression. Dedicated early pregnancy units (EPUs) are currently being developed in several countries around the world (for example, the US, the UK, Canada, Ireland, and Australia) to provide optimal medical and psychological management and continued support. This type of unit could help to meet the needs of women with high levels of stress during the first trimester. A recent study by Coomarasamy *et al* showed that women who received care in dedicated EPUs were satisfied with their management, and the authors thus encourage healthcare funders and providers to invest in early pregnancy care.^17^ A study by de Montigny *et al* showed that SM also has psychological repercussions for the father, particularly sadness, sometimes accompanied by emotional detachment during a subsequent pregnancy for fear of experiencing the same event again.^7^ The impact on men is likely underestimated even more than in women. It would thus be interesting to evaluate the impact of prior SM on the father’s level of stress during a subsequent pregnancy.^25^

In conclusion, this study has shown a significant association between a history of SM and a higher level of stress in pregnant women during the first trimester of a subsequent pregnancy. Additional insights could be provided by an investigation of the association between prior SM and anxiety or depression during subsequent pregnancies. Further research is also warranted into the optimal management of stress and psychological repercussions during early pregnancy in women with a history of SM.

## Data

The dataset relied on in this article is available from the corresponding author on reasonable request.
